# Oscillatory neurofeedback networks and poststroke rehabilitative potential in severely impaired stroke patients

**DOI:** 10.1016/j.nicl.2022.103289

**Published:** 2022-12-14

**Authors:** Kevin Kern, Mathias Vukelić, Robert Guggenberger, Alireza Gharabaghi

**Affiliations:** Institute for Neuromodulation and Neurotechnology, University of Tübingen, Germany

**Keywords:** Brain-robot interface, Brain-computer interface, Long-range connectivity, Phase synchronization, Neurorehabilitation, Motor restoration

## Abstract

Motor restoration after severe stroke is often limited. However, some of the severely impaired stroke patients may still have a rehabilitative potential. Biomarkers that identify these patients are sparse.

Eighteen severely impaired chronic stroke patients with a lack of volitional finger extension participated in an EEG study. During sixty-six trials of kinesthetic motor imagery, a brain-machine interface turned event-related beta-band desynchronization of the ipsilesional sensorimotor cortex into opening of the paralyzed hand by a robotic orthosis. A subgroup of eight patients participated in a subsequent four-week rehabilitation training. Changes of the movement extent were captured with sensors which objectively quantified even discrete improvements of wrist movement. Albeit with the same motor impairment level, patients could be differentiated into two groups, i.e., with and without task-related increase of bilateral cortico-cortical phase synchronization between frontal/premotor and parietal areas. This fronto-parietal integration (FPI) was associated with a significantly higher volitional beta modulation range in the ipsilesional sensorimotor cortex. Following the four-week training, patients with FPI showed significantly higher improvement in wrist movement than those without FPI. Moreover, only the former group improved significantly in the upper extremity Fugl-Meyer-Assessment score.

Neurofeedback-related long-range oscillatory coherence may differentiate severely impaired stroke patients with regard to their rehabilitative potential, a finding that needs to be confirmed in larger patient cohorts.

## Highlights

• Markers for rehabilitative potential of severely impaired stroke patients are sparse.
• Neurofeedback with a robotic orthosis induced cortical alpha synchronization.
• Fronto-parietal synchronization differentiated patients with the same hand paralysis.
• This synchronization determined ipsilesional sensorimotor beta modulation.
• This synchronization distinguished patients with regard to the restorative potential.

## 1 Introduction

About one third of stroke patients show no spontaneous motor recovery [Prabhakaran et al., 2008, Winters et al., 2015]. The lack of spontaneous recovery and the initial severity after stroke lead to poor motor recoverers with severe motor impairment; they are characterized by the absence of finger extension within 72 h poststroke [Winters et al., 2015] and a severe degeneration of corticospinal integrity [Byblow et al., 2015, Buch et al., 2016]. When considering this patient group as homogenous with regard to their low potential to recover at the impairment level, the choice for therapy may be guided towards training compensation strategies and not improving function [Winters et al., 2015]. From a neurophysiological perspective, however, these poor motor recoverers may also be considered quite heterogeneous [Belardinelli et al., 2017]. Therefore, some of them may be potential candidates for physiologically-informed interventions that target residual cortico-cortical networks and cortico-spinal pathways to achieve at least partial restitution of motor function [Gharabaghi, 2016]. In this context, reliable and physiologically plausible biomarkers are necessary to support the investigation of restorative interventions in this patient population. Task-related cortical oscillatory activity may provide such a biomarker.

During motor execution and imagery, sensorimotor beta oscillations show typical patterns of event-related desynchronization (ERD) and synchronization (ERS) [Pfurtscheller and Lopes da Silva, 1999]. They enhance signal propagation in the motor system [Romei et al., 2016] and determine the input–output ratio of corticospinal excitability in a frequency- and phase-specific way [Khademi et al., 2018, Khademi et al., 2019, Naros et al., 2019]. Furthermore, the movement-related beta ERD/ERS modulation range (summarized as event-related spectral perturbation, ERSP) is compromised in stroke patients proportionally to the motor impairment level, thereby providing a plausible physiological target for therapeutic interventions [Rossiter et al., 2014, Shiner et al., 2015]. However, capturing movement-related beta ERSP is challenging in patients with severe and persistent motor deficits who are unable to extend the affected fingers. In these patients another approach is necessary for eliciting ERSP than in patients with residual movement. Motor imagery (MI) and neurofeedback by using a brain-computer/brain machine interface may provide such an alternative approach. Specifically, MI and contingent proprioceptive feedback – rather than visual feedback only - were able to increase the range, accuracy and duration of self-regulated beta modulation and facilitated learning volitional control of this sensorimotor oscillatory pattern in healthy subjects [Vukelić and Gharabaghi, 2015a, Naros et al., 2016a, Naros et al., 2016b, Darvishi et al., 2017]. Notably, the self-regulated beta modulation range proportionally enhanced the subsequent corticospinal excitability [Kraus et al., 2016a, Royter and Gharabaghi, 2016] and motor improvement [Naros et al., 2016a, Naros et al., 2016b], thereby inspiring pilot studies to take the same approach in stroke patients [Naros and Gharabaghi, 2015, Belardinelli et al., 2017, Khademi et al., 2022]. These neurofeedback tasks can, however, be challenging even for healthy subjects [Fels et al., 2015] and particularly for severely impaired stroke patients with a compromised modulation range [Gomez-Rodriguez et al., 2011a, Gomez-Rodriguez et al., 2011b, Gomez-Rodriguez et al., 2011b, Brauchle et al., 2015]. Along these lines, the activation of a distributed oscillatory network suggests that MI and proprioceptive feedback is a cognitively demanding task [Vukelić et al., 2014, Vukelić and Gharabaghi, 2015a, Vukelić and Gharabaghi, 2015b]. Specifically, in healthy subjects good and poor performers of sensorimotor beta self-regulation could be differentiated on the basis of their task-related cortical network activity which was estimated via the phase slope index [PSI, Nolte et al., 2008] in electroencephalographic recordings during MI-related brain-machine interface control of a robotic hand orthosis [Vukelić et al., 2014]. The analysis of effective connectivity revealed that a fronto-parietal information flow in the oscillatory alpha band is associated with the sensorimotor beta modulation range [Vukelić et al., 2014].

Here, we applied the very same approach in severely impaired chronic stroke patients with a lack of volitional finger extension. Specifically, MI and proprioceptive feedback via a brain-machine interface-controlled robotic orthosis were used as an intervention for poststroke rehabilitation. Before this four-week intervention, we applied a single session of the very same task to predict the rehabilitative potential. Importantly, in healthy subjects, good and poor performers of this cognitively demanding task could be differentiated on the basis of their task-related cortical network activity in electroencephalographic recordings. Therefore, cortical networks and local power modulations may index different things and indicate different rehabilitation types needed. We hypothesized that task-related cortico-cortical connectivity in a fronto-parietal alpha-band network in severely impaired stroke patients (i) determines their performance of sensorimotor beta-band self-regulation and (ii) differentiates this patient population with regard to the restorative potential following the four-week intervention.

## 2 Methods

### 2.1 Patients and overview

Having given their written informed consent, eighteen stroke patients (13 males, mean age = 60.4 ± 9.9 [34 72] years) were recruited for this study which was approved by the local ethics committee. Patients had a subcortical, cortical or mixed (subcortical and cortical) stroke affecting the motor pathway. They were at least seven months post stroke (mean time = 81.1 ± 54.4 [8 244] months) and showed severe and persistent motor deficits in the upper limb (mean upper extremity Fugl-Meyer-Assessment (UE-FMA) score: 16.39 ± 5.94 [6.80 28.60]). None of the patients were able to voluntarily extend the fingers of their affected hand.

In this study, a neurofeedback task was applied using a brain-computer/brain-machine interface (BCI/BMI) set-up. Due to the large overlap between the neurofeedback concept and the BCI/BMI methodology in the literature, the terminology from both fields is used throughout the manuscript. In short, the task consisted of kinesthetic motor imagery of opening the paralyzed hand and contingent, i.e., non-continuous, proprioceptive neurofeedback. We used the EEG-recorded power decrease in the beta frequency band as the feedback target and a BMI-controlled robotic hand orthosis to deliver feedback in the form of finger opening. The fact that the patients saw the hand opening introduced visual elements to the proprioceptive feedback and could also affect the kinesthetic motor imagery paradigm through a positive reinforcement loop.

This neurofeedback task was used both during one screening session and during a four-week rehabilitation training. During the screening session EEG connectivity was estimated and compared to the outcome after the rehabilitation training. Previously, EEG connectivity instead of power measures have been shown to better differentiate between kinesthetic and visual MI. Moreover, the connectivity patterns in this previous work were correlated between kinesthetic MI and motor execution, and between visual MI and visual observation (Yang et al., 2021). These findings support the use of kinesthetic MI in the context of motor rehabilitation and connectivity analysis for the prediction of the rehabilitative potential as applied in this study.

The following aspects will be described in detail in the subsequent chapters:

• Neurofeedback task with ipsilesional sensorimotor beta power changes as the feedback target and the BMI setup with a robotic hand orthosis providing proprioceptive feedback via finger movement.
• Preprocessing of the EEG data acquired during this neurofeedback task in the screening session.
• Estimation of the beta power modulation in the ipsilesional sensorimotor cortex during the screening session.
• Estimation of cortico-cortical effective connectivity during the screening session using the phase slope index (PSI).
• Calculating the stability if this connectivity and showing the direction of information flow between all edges of the network.
• Estimating the association of fronto-parietal network connectivity and ipsilesional beta power modulation during the screening session.
• A subgroup of patients participated in a four-week intervention including the neurofeedback task with a clinical assessment (UE-FMA) at the start and the end of the training period (Follow-up: Rehabilitation training).
• At the start and the end of the training period, also a sensor-based assessment of the maximum range of motion of wrist extension and flexion was performed (Follow-up: Objective assessment).

### 2.2 Task and setup: beta-ERD based proprioceptive neurofeedback

All patients were comfortably seated upright in a chair. Scalp EEG potentials were recorded (BrainAmp, Brainproducts GmbH, Germany) at the following 32 positions of the international 10–5 system: Fp1, Fp2, F3, Fz, F4, FT7, FC5, FC3, FC1, FC2, FC4, FC6, FT8, C5, C3, C1, Cz, C2, C4, C6, TP7, CP5, CP3, CP1, CPz, CP2, CP4, CP6, TP8, P3, P4, POz (Ag/AgCl electrodes, FCz was used as common reference, grounded to AFz). All impedances were kept below 10 kΩ at the onset of each session. EEG data was digitized at 1 kHz, high pass filtered at 0.1 Hz, transmitted to the BCI2000 software [Schalk et al., 2004] for on-line processing and then stored for off-line analysis.

The patients’ range of ipsilesional beta (β)-modulation induced by MI was assessed in a task combined with contingent proprioceptive/haptic neurofeedback, i.e., robot-assisted movements. Patients were instructed to use kinesthetic (first person perspective) MI of opening their affected hand [Neuper et al., 2005], which was attached to a robotic hand orthosis (Amadeo® system, Tyromotion GmbH, Austria). MI resulted in an event-related decrease (ERD) of β-power over the ipsilesional sensorimotor cortex (FC4, C4 and CP4) which controlled a brain-machine interface to passively open the paralyzed hand by a hand orthosis. When MI was discontinued, indicated by increased β-power, the finger extension ceased. At the end of the move phase, the hand was returned to the starting position.

**Fig. 1:**
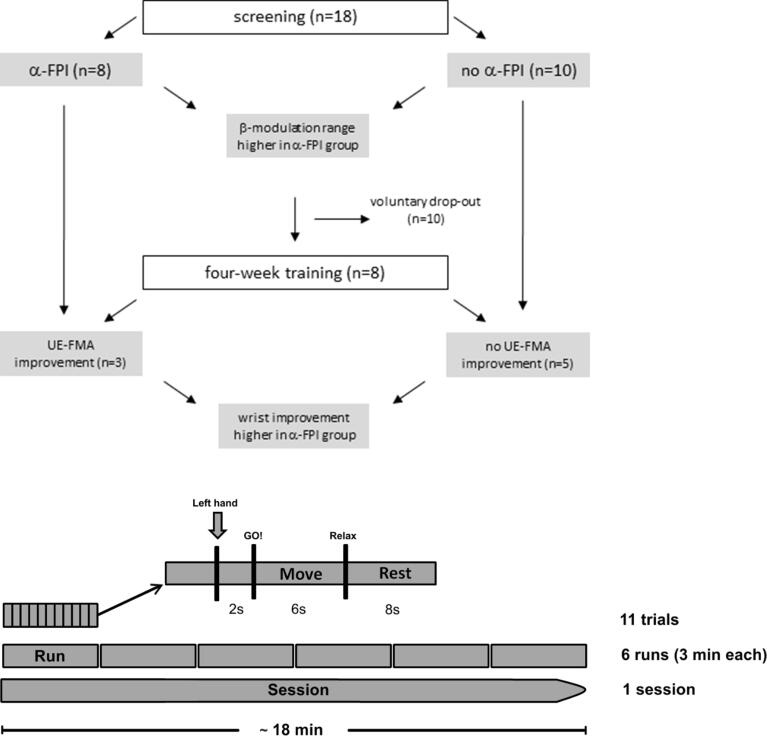
**Experimental design. Upper panel: Study flow diagram and summary of findings.** Eighteen severely impaired chronic stroke patients with a lack of volitional finger extension participated in one screening session of kinesthetic motor imagery and brain-machine interface (BMI)-controlled feedback with robotic orthosis opening the paralyzed hand. A subgroup of eight patients participated in a subsequent four-week rehabilitation training. Albeit with the same motor impairment level, patients could be differentiated into two groups, i.e., with and without BMI task-related increase of bilateral cortico-cortical phase synchronization between frontal/premotor and parietal areas. This fronto-parietal integration (FPI) was associated with a significantly higher volitional beta modulation range in the ipsilesional sensorimotor cortex. Following the four-week training, patients with FPI showed significantly higher improvement in wrist movement than those without FPI. Moreover, only the FPI group improved significantly in the upper extremity Fugl-Meyer-Assessment score. **Lower panel: Time course of the experimental paradigm during screening** Patients were instructed to use kinesthetic motor imagery (MI) of opening their affected hand, which was attached to a robotic hand orthosis. A trial consisted of three phases with 2 *sec* preparation, 6 *sec* kinesthetic MI of hand opening and 8 *sec* relaxation. The preparation phase was cued by the auditory command “hand”, before the “Go” cued MI of hand opening. MI resulted in an event-related decrease (ERD) of β-power over the ipsilesional sensorimotor cortex (FC4, C4 and CP4) which controlled via a brain-machine interface the robotic hand orthosis, i.e., providing contingent proprioceptive neurofeedback. When MI was discontinued, indicated by increased β-power, the robot-assisted finger extension ceased. At the end of the move phase, the robotic hand orthosis was returned to the starting position.

The patients performed one session of MI of opening the affected hand which consisted of 6 runs, each of which lasted 3 min (Fig. 1). To control for muscle contraction, bilateral hand and forearm electromyographic (EMG) activity was monitored online for Flexor Carpi Radialis (FCR), Extensor Carpi Radialis (ECR), Extensor Digitorum Communis (EDC) and Extensor Carpi Ulnaris (ECU) muscles. A run consisted of 11 trials, each of which was divided into three phases (2 *sec* preparation, 6 *sec* kinesthetic MI of hand opening, 8 *sec* relaxation) while each phase was initiated by an auditory cue. For online classification, oscillatory power was estimated every 40 ms over the last 500 ms using an autoregressive model based on the Burg Algorithm with a model order of 16 [McFarland and Wolpaw, 2008]. A linear threshold-based classifier of 9 features (3 channels [FC4/C4/CP4] × 3 2-Hz frequency bins (16–22 Hz) was used to detect a decrease in β-power. In our previous work in healthy subjects and stroke patients, this frequency band has been shown to be particularly suitable for integrating motor imagery and proprioceptive feedback. Thus, our linear threshold-based classifier detected a drop in β-power relative to the average of the last 10 s of the other phases (rest & preparation). Additionally, we adapted the threshold of the classifier to maximize patients’ individual modulation of β-power. An initial run of 3 min immediately prior to the patients performing the training session was used to estimate the threshold of the classifier with the maximal possible predictive value, i.e., the highest link with β-modulation. On the basis of this estimation, we introduced an offset parameter into the classifier to maximize the patient-specific modulation of β-power [Bauer and Gharabaghi, 2017].

### 2.3 Screening data: pre-processing

All runs were grouped together, resulting in a data set of 18 min of EEG data per patient. We excluded eight EEG channels (Fp1, Fp2, FT7, FT8, C5, C6, TP7, and TP8) from offline-analysis due to artifact contamination, e.g., related to eye and jaw movements and/or high impedances. We divided the whole data set of 18 min into non-overlapping epochs of 4 s duration. Epochs were rejected if they contained a maximum deviation above 60 µV in any of the EEG channels [Sanei, 2007] or if activity in the non-affected hand showed relevant EMG activity. The EEG signals were detrended, zero-padded and low-pass filtered up to 48 Hz for calculation of stability of network activity across frequencies. A filter of 6 to 16 Hz was chosen for calculation of connectivity in the alpha (α)-frequency range. For calculation of event-related spectral perturbation (ERSP), signals were band-pass filtered between 14 and 24 Hz. All filtering was performed with a first order zero-phase lag FIR filter.

### 2.4 Screening data: beta modulation range in the ipsilesional sensorimotor cortex

**Fig. 2:**
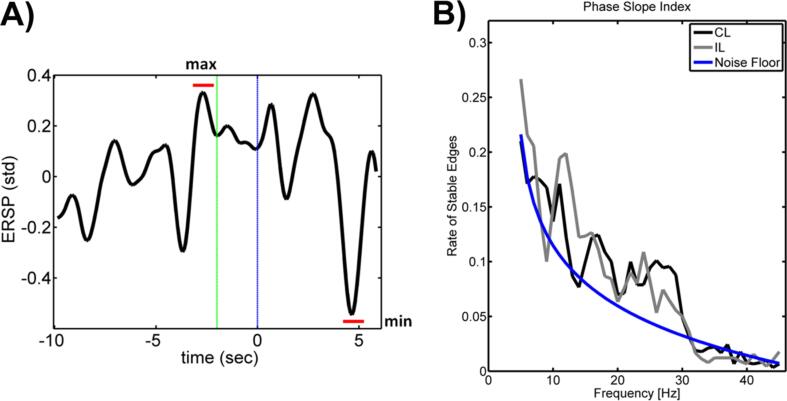
**A) Definition of the β-modulation range:** The plot shows the time course of the event-related spectral perturbation (ERSP) of one representative patient during the task. The abscissa represents the time axis, with the rest phase from −10 to −2 *sec* (green line), the preparation phase from −2 to 0 *sec* (blue line), and the kinesthetic motor imagery phase from 0 to 6 *sec*. The ERSP is averaged across trials. The value is visualized on a standard deviation scale, and normalized with respect to the rest baseline. The β-modulation range is defined as the individual maximum difference between the minimum in the kinesthetic motor imagery phase and the maximum in the rest phase in the beta range (16–22 Hz). **B) Stability of network activity across frequencies:** The plots show the number of significant edges of effective connectivity (phase slope index) separated from the noise floor and averaged for every frequency across all patients. The black line shows stable edges at contralesional (CL) hemisphere, the gray line shows stable edges at ipsilesional (IL) hemisphere. The blue line represents the estimated noise floor as explained by a 1/f noise model. At both hemispheres, the maximum elevation over the noise floor of network stability is located in the α-frequency range (between 8 and 14 Hz). (For interpretation of the references to color in this figure legend, the reader is referred to the web version of this article.)

We calculated offline the ERSP between 16 and 22 Hz with a frequency resolution of 0.24 Hz, as implemented in the EEGLab toolbox [Delorme and Makeig, 2004]. This map was averaged across the ipsilesional sensorimotor electrodes (FC4, C4 and CP4) for each frequency bin. As a performance measure, we calculated patient’s individual β-modulation range. Hence, we selected the individual frequency bin with the maximum difference between the ERSP *minimum* in the kinesthetic MI phase (i.e., the potential to maximally desynchronize during the kinesthetic MI phase) and the ERSP *maximum* in the rest phase (i.e., the potential to maximally synchronize during the rest phase). Fig. 2A illustrates how the β-modulation range was defined on the basis of the ERSP in a representative patient. The ERSP was calculated run-wise (rest, preparation, kinesthetic MI) for 16 *sec* and averaged across runs for each patient. This value was normalized with respect to the rest period and visualized across time with −10 to −2 *sec* of rest phase, −2 to 0 *sec* of preparation phase, and 0 to 6 *sec* of kinesthetic MI phase. Finally, the correlation coefficient (Pearson and Spearman) was calculated between the ERSP and the UE-FMA score.

### 2.5 Screening data: estimation of cortico-cortical effective connectivity

The estimation of the phase slope index (PSI) is based on the coherency function [Nolte et al., 2008]. Each unrejected epoch was subdivided into segments of 1 *sec* length with 50 % overlap, corresponding to a frequency resolution of δf = 1 Hz. Each segment was multiplied with a Hanning window. We chose the frequency range of 8–14 Hz to estimate the α-network activity. A Fourier transformation of the data resulted in an estimation of the cross-spectra between two time series. PSI indicates synchronization of two processes which are time-lagged to each other [Nolte et al., 2008]. PSI was defined as

$${\mathrm{PSI}}_{\mathrm{ij}}\left(f\right)=I\left(\sum_{f\;\in F}C_{ij}^{*}\left(f\right)C_{ij}\left(f+\;\delta f\right)\right),$$

where $C_{\mathrm{ij}}$ was the complex coherency between channels *i* and *j*, δf the frequency resolution, $I\left(∙\right)$ the imaginary part and *f* the frequency band of interest used to calculate the slope. PSI estimates were obtained from each unrejected epoch. Furthermore, we calculated net information flow for the *ith* channel defined as

$${\mathrm{PSI}}_{\mathrm{sum}}\left(i,f\right)=\sum_{j}{PSI}_{ij}\left(f\right).$$

where ${PSI}_{sum}$ > 0 denoted net drivers and ${PSI}_{sum}$ < 0 net recipient. The connectivity measure was calculated using scripts written or adapted in MATLAB®.

## 3 Screening data: Calculation of network stability

PSI is a signed quantity with a null hypothesis of zero mean, and only the sign of PSI is independent from the power of the signal [Haufe et al., 2013]. Hence, we tested for non-zero median with a sign-test which is more robust despite power fluctuations and provides a straightforward interpretation, even in the absence of normality. We therefore assessed the stability of networks over all unrejected epochs on the patient level. Significance threshold was set to p ≤ 0.01. This meant that an edge (PSI) or electrode (PSIsum) is likely to be a stable sender or receiver, indicating a sending direction which is persistent and consequently activated during the task. A network is therefore considered stable, if a cortico-cortical connection with a stable direction of sending can be detected during the task. To establish frequencies with stable networks during the modulation of ipsilesional β-oscillations, we averaged the number of significant edges (PSI) over all patients for every frequency for the ipsilesional (IL) and contralesional (CL) hemispheres indicating the amount of stability in sending directions in each hemisphere (possible results: sender, receiver or no connection). Furthermore, we separated the network stability from the noise floor as described by a 1/f noise model [Blankertz et al., 2010]. For analysis on group level, we utilized binomial statistics to estimate the probability that the observed stable edge (PSI) or electrode (PSI_sum_) would have occurred with alpha-error likelihood (p ≤ 0.05, Bonferroni-corrected for multiple comparison).

## 4 Screening data: association of cortico-cortical α-network activity and ipsilesional β-modulation

Between frontal (F4/F3) / premotor (FC2/FC4/FC6/FC1/FC3/FC5) regions and parietal / parieto-occipital regions (P4/POz/P3/POz), there where altogether 32 possible edges for both hemispheres. For each patient, the number of significant edges was divided by this maximum number (i.e., 32) of possible edges, resulting in a FPI value between 0 and 1. We then classified the patients in two groups: patients with no significant edges (i.e., 0) were assigned to the group “without FPI”, whereas patients with at least one connection (i.e., >0) were assigned to the group “with FPI”.

We then used a two-sample *t*-test to establish how FPI influenced the β-modulation range. This approach was based on previous findings in severely impaired stroke patients that volitional modulation of ipsilesional neural activity controlling a brain–machine interface (similar to the one applied in this study) relies on structural and functional connectivity in both ipsilesional and contralesional fronto-parietal pathways (Buch et al., 2012). While all patients in this study showed significant connections between some brain areas, e.g., involving sensorimotor regions, only a subgroup also showed long-range connections between frontal and parietal regions. We considered all patients with no such long-range connectivity between frontal and parietal regions as lacking FPI.

### 4.1 Follow-up: rehabilitation training

A subgroup of eight patients (7 males, mean age: 58.8 ± 11.5 [34 68] years; 75.6 ± 36.8 [34 156] months after stroke; mean UE-FMA score of 16.64 ± 6.48 [6.80 28.60]) decided to also take part in a subsequent training program. Patients were evaluated with the UE-FMA before and after the intervention; these assessments were videotaped. Five independent raters evaluated the video tapes and were blinded with regard to the time point of assessment (pre vs post training). The patient characteristics are summarized in Table 1. The patients were part of a larger rehabilitation study and participated in 20 intervention sessions over a period of four weeks. Each session consisted of brain self-regulation and feedback via proprioceptive stimulation with a hand robot (approximately 150 trials per day; [Naros and Gharabaghi, 2015]) prior to a movement training with an exoskeleton attached to the impaired arm (approximately 150 trials per day; Grimm et al., 2016b). A detailed description of the therapy concept and intervention applied in this study is set out in previous work [Belardinelli et al., 2017, Gharabaghi, 2016]. Seven of the patients (P1-P7) were previously reported with regard to changes of cortico-muscular coherence during and following the intervention period [Belardinelli et al., 2017, Khademi et al., 2022]. In this study, they were studied with regard to the role of cortico-cortical oscillatory networks before the intervention period. Two of the patients (P2 and P3) have been described elsewhere with respect to the evolution of their whole upper extremity kinematic parameters in the course of the intervention [Grimm et al., 2016a].

**Table 1:** Patient characteristics.

P	Age	Gender	Hand	FPI	Lesion location	Stroke Type	MS	WMP		UE-FMA	
								*start*	*end*	*start*	*end*
P1	56	M	right	yes	mixed	H	78	46.5	103.1	28.6	32.25
P2	63	F	right	yes	mixed	I	78	22.3	36.1	16.1	18.35
P3	52	M	right	yes	cortical	I	156	34.8	66.1	22.4	26.45
P4	67	M	right	no	mixed	H	75	1.6	18.1	6.8	10.85
P5	68	M	right	no	subcortical	H	34	60.7	72.2	16.4	20.2
P6	34	M	right	no	mixed	H	45	18.7	18.1	13.4	13.4
P7	63	M	right	no	mixed	I	58	4.5	4.6	15.8	16.8
P8	67	M	left	no	mixed	I	81	5.4	13.4	13.6	13.4

P (patient number), age (years), gender (M = male, F = female), FPI (group affiliation with or without FPI), lesion location, stroke type (H = hemorrhagic, I = ischemic), months after stroke (MS), average wrist movement performance values (WMP in degrees), and averaged upper extremity Fugl-Meyer-assessment score (UE-FMA) of different raters.

### 4.2 Follow-up: objective assessment

The UE-FMA evaluation was supplemented by a sensor-based assessment of the maximum range of motion of wrist extension and flexion in joint space using an attached upper limb exoskeleton [Kern et al., 2021]. We selected wrist movements for this evaluation, since this was the most distal range of motion that could be assessed with the exoskeleton in this patient group that was characterized by the paralyzed hand. The exoskeleton-based assessment applied here provides convergent validity with respect to the UE-FMA score in severely impaired stroke patients [Grimm et al., 2021]. The device was calibrated according to the individual anatomy (e.g., shoulder position, forearm/upper arm length) of each patient. The general experimental set-up has already been described in detail elsewhere [Grimm et al., 2016a, Grimm et al., 2016b, Grimm et al., 2016c, Grimm and Gharabaghi, 2016] and is cited here when applied in the same way: We used a commercially available (Armeo Spring, Hocoma, Volketswil, Switzerland, Fig. 1, right side) rehabilitation exoskeleton for shoulder, elbow and wrist joints to provide antigravity support for the paretic arm and registration of movement kinematics and grip force. This device enabled us to make individual adjustments of gravity compensation, thereby supporting patients with severe impairment in harnessing even minimal volitional motor control. This set-up also enabled us to capture the improvement of even discrete movements with high accuracy [Grimm et al., 2016a, Grimm et al., 2016b, Grimm and Gharabaghi, 2016, Grimm et al., 2021]. Specifically, kinematic sensor data was provided by built-in angle sensors (sensor resolution < 0.2°). In the present study, we used the data acquired by the wrist flexion/extension sensor, while the rest of the upper limb was positioned in a relaxed position in front of the patient, i.e., shoulder adduction, 90-120° elbow flexion, wrist in upright position between pronation and supination. Each trial had visual and auditory cues and consisted of a “rest”, “wrist extension” and “wrist flexion” phase, each lasting 5 s. The patients were instructed to extend and flex their wrist as far as possible during each trial. All other upper limb joints of the exoskeleton were blocked during these trials to reinforce pure wrist movement and avoid compensatory movements in other joints. The wrist movement performance (WMP) was calculated as the maximum angle in degrees, i.e., the difference between maximum extension and maximum flexion as captured by the exoskeleton sensor. The WMP improvement following the four-week training program was calculated as the average change from the first to the last 40 trials. A one-sample *t*-test was used to estimate the significance of the training induced changes (WMP and UE-FMA) from the start to the end of the four-week intervention for the entire group of eight patients, and for the subgroups with and without FPI. A two-sample *t*-test was used to compare these changes between the subgroups (with and without FPI).

## 5 Results

Both hemispheres revealed that stable effective connectivity networks are present during the task. The maximum average elevation over the noise floor level occurred in the α-range (between 8 and 14 Hz) and decreased with increasing frequency from β- to γ- range (Fig. 2B).

**Fig. 3:**
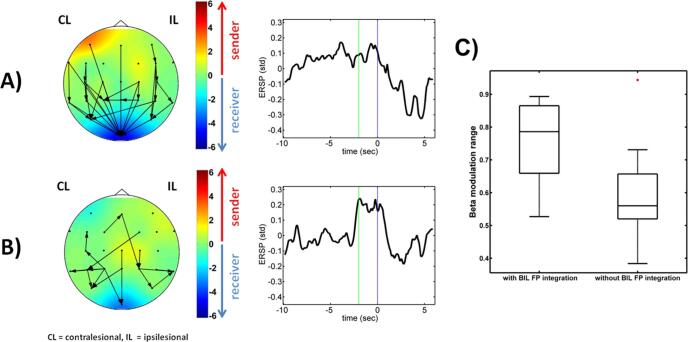
**Patients with high and low β-modulation range have different stable effective connectivity (PSI_sum_/PSI) networks in the α-frequency range and different contrasts of β-modulation during the task: A) and B)** shows patients with fronto-parietal integrity (N = 8) and without fronto-parietal integrity, i.e., no significant long-range connections between frontal and parietal regions (N = 10), respectively. The left column shows the stable effective connectivity networks (PSI_sum_ /PSI) in the α-frequency range. Cortical information flow [PSI, arrows indicate significant connections] and net cortical information flow are depicted [PSI_sum_ topoplot, numbers on the scale indicate a logarithmic visualization of the p-values, e.g., for p = 0.01: |log(0.01)| = 2]. The red color indicates net drivers and the blue color indicates net recipients. The middle column shows the time course of the event-related spectral perturbation (ERSP) during the task. The abscissa represents the time axis, with the rest phase from −10 to −2 *sec* (green line), the preparation phase from −2 to 0 *sec* (blue line), and the kinesthetic motor imagery phase from 0 to 6 *sec*. The ERSP was averaged across trials on an individual level and have been averaged across patient’s individual maximum β-modulation range on a group level (visualized on a standard deviation scale, and normalized with respect to the rest baseline). **C) Patients with bilateral fronto-parietal integrity have a higher β-modulation range:** Boxplots of the β-modulation range for patients with (left) and without (right) bilateral (BIL) fronto-parietal integration (FP-I) Patients showing bilateral fronto-parietal integration have a significantly higher β-modulation range (two-sample *t*-test, p = 0.038). (For interpretation of the references to color in this figure legend, the reader is referred to the web version of this article.)

Patients were classified into two groups, i.e., with (n = 8) and without any (n = 10) significant edges of FPI, i.e., alpha-band information flow between frontal/premotor and parietal areas (Fig. 3A/B left); with FPI being associated with a significantly higher volitional beta modulation range in the ipsilesional sensorimotor cortex (*t*-test, dof = 16, p < 0.05) (Fig. 3C). This finding was independent of other patient characteristics. In particular, the beta modulation range and the UE-FMA score were not correlated (Pearson’s correlation coefficient, r = −0.06, p = 0.8, Spearman's rank correlation coefficient, r = −0.18, p = 0.5).

The groups with and without FPI also differed with regard to their ERSP morphology (Fig. 3 A/B middle). As for their sensorimotor beta modulation range, good and poor performers showed prominent desynchronization in the MI phase and synchronization in the preparation phase, respectively.

The subgroup of patients (n = 8) participating in the four-week intervention (including patients with and without FPI) improved significantly with regard to the WMP from 24.3 ± 21.5 [1.6 60.7] to 41.5 ± 35.1 [4.6 103.1] and the UE-FMA from 16.64 ± 6.48 [6.80 28.60] to 18.96 ± 7.24 [10.85 32.25] (one-sample *t*-test, p = 0.037 and p = 0.0088, respectively). The motor improvement for each individual subject is specified in Table 1. The improvement in wrist movement was significantly higher (two-sample *t*-test, dof = 6, *t*-score = 2.66, p = 0.038) in patients with FPI. Moreover, only the FPI group (but not the group with no FPI) showed a significant improvement in the UE-FMA score following training (one-sample *t*-test, p = 0.026), thereby, determining the motor improvement observed for the whole group.

## 6 Discussion

This study provided novel findings with regard to the task-related cortical connectivity of severely impaired chronic stroke patients:

1. Kinesthetic motor imagery and proprioceptive neurofeedback differentiated stroke patients with hand paralysis with regard to their cortical synchronization profile, i.e., the presence of fronto-parietal connectivity.
2. Bilateral fronto-parietal phase synchronization in the alpha band might influence the ipsilesional sensorimotor beta modulation range.
3. This fronto-parietal integration differentiated the patients with regard to their restorative potential as measured by wrist angle movement and a clinical score.

### 6.1 Fronto-parietal integration and sensorimotor modulation range

In previous studies by other groups, poor motor recoverers were also characterized by lower initial EEG functional connectivity (FC) between the affected hemisphere and the rest of the brain than patients with proportional recovery; furthermore, the former showed a degeneration of corticocortical fiber tracts in the subsequent months after stroke [Guggisberg et al., 2017]. These observations were in line with other findings in chronic stroke patients: Higher structural connectivity of fronto-parietal pathways was related to more residual motor function [Schulz et al., 2015] and to better volitional control of sensorimotor rhythms [Buch et al., 2012], in mildly/moderately and severely impaired patients, respectively.

On the other hand, functional connectivity analysis of oscillatory network interactions in mildly/moderately affected patients painted a more diverse picture: Motor cortex connectivity in the task-free condition evolved dynamically over time, while higher coherence correlated with both the functional improvement and impairment level in the course of poststroke recovery [Westlake et al., 2012, Dubovik et al., 2012, Nicolo et al., 2015]. Furthermore, higher fronto-parietal connectivity during a visuomotor grip task was associated with a higher residual motor deficit, possibly reflecting either maladaptive or adaptive mechanisms, i.e., the cause for the functional impairment or the (hitherto unsuccessful) attempt to generate motor output, respectively [Bönstrup et al. 2018].

The present work contributed to this question by investigating patients with severe and persistent motor deficits of the upper limb who were unable to voluntarily extend the fingers of their affected hand. The behaviorally relevant beta modulation range in relation to cortico-cortical alpha coherence was probed during MI of finger extension and contingent proprioceptive neurofeedback. This ensured that the detected cross-frequency association was independent of the clinical status since the impaired transmission along the efferent pathway in the corticospinal tract to the paralyzed fingers was the common denominator in all patients. In this context, the present study revealed a task-related association between long-range cortico-cortical alpha synchronization and sensorimotor beta modulation in the severely lesioned brain. While it is generally accepted that lower EEG frequencies correspond to distributed brain activity over larger spatial regions, in motor networks this is not always the case. Alpha and beta rhythms have been related to local information processing and long-range connections, respectively [Athanasiou et al. 2018]. In the poststroke brain, further adaptations may occur with regard to the involved local and long-range frequency spectrum. Specifically, the cortical power modulations and long-range cortico-muscular connectivity changes included both the alpha and beta frequency bands, thereby, suggesting a different mode of poststroke sensorimotor processing than in healthy subjects [Khademi et al. 2022].

The enhanced fronto-parietal integration occurred in the patient subgroup that showed a higher beta modulation range and physiological ERSP morphology with a prominent desynchronization in the MI task phase. This observation suggests that such a cortical activation pattern represented a specific network-adaptation – by way of a mediating mechanism - related to the visuomotor and sensorimotor task demands and that it is not merely a property of the stroke-lesioned brain [Bönstrup et al. 2018]. Accordingly, previous work of our group in healthy subjects who performed the same neurofeedback task suggests that this long-range synchronization may reflect task-related cognitive demands [Vukelić et al., 2014, Fels et al., 2015]. Consistently, patients with the limited beta modulation range also showed a more diffuse cortico-cortical connectivity pattern and somewhat atypical beta synchronization in the preparatory task phase. This might reflect anticipatory up-regulation of attention in the sensorimotor system before the MI phase [Kilavik et al., 2013]. The reason for these differences between good and poor performers remains unclear. Future analyses of lesion area or tractography may help to better understand the underlying mechanisms.

### 6.2 Differentiating subgroups of stroke patients with hand paralysis

The field of restorative research in severely impaired patients has to face specific challenges: (i) The effect sizes of motor improvement at the impairment level (UE-FMA) will, per definition, be low for these severely affected chronic patients and may not achieve the clinically important difference (CID) for the UE-FMA score (i.e., 5.25) that has been estimated for patients with minimal to moderate impairment (UE-FMA score of ≥28 to ≤50; [Page et al., 2012]). (ii) In addition, poor recoverers may respond differently to rehabilitation interventions. (iii) Therapy effects at the sub-clinical level may be overlooked. (iv) Novel interventions may be prematurely dismissed as ineffective. The present work indicated that poor motor recoverers [Prabhakaran et al., 2008, Winters et al., 2015] may be classified in subgroups on the basis of cortico-cortical phase synchronization during ipsilesional sensorimotor self-modulation. Notably, this stratification was possible in patients with hand paralysis by applying motor imagery and proprioceptive feedback. We acknowledge that motor imagery alone (i.e., without neurofeedback) could have caused the same or similar results. However, this approach extended previous investigations on the movement-related beta modulation range in mild to moderately impaired patients in whom a higher modulation range corresponded to less motor impairment [Rossiter et al., 2014, Shiner et al., 2015].

We speculate that the good performers in the applied task, i.e., those with a higher beta modulation range and bilateral cortico-cortical phase synchronization, showed a higher sustainability for repetitive training and were therefore more liable to show clinical improvements. The poor performers of the present study with the restricted beta modulation range – if considered for restorative interventions – would need to be addressed differently; potential strategies include: (i) Optimized neurofeedback algorithms to overcome cognitive load issues, maintain motivation and improve reinforcement learning [Bauer and Gharabaghi, 2015a, Bauer and Gharabaghi, 2015b, Bauer et al., 2015, Bauer et al., 2016a, Bauer et al., 2016b, Guggenberger et al., 2022]. (ii) Augmentation of feedback with multi-joint exoskeletons and virtual reality [Grimm et al., 2016a, Grimm et al., 2016b]. (iii) Enhancement of the cortical modulation range by concurrent cortical [Naros and Gharabaghi, 2017] and/or neuromuscular electrical stimulation [Grimm and Gharabaghi, 2016, Grimm et al., 2016c]. (iv) Signal detection closer to the source by epidural interfaces [Gharabaghi et al., 2014b, Gharabaghi et al., 2014c, Spüler et al., 2014].

### 6.3 Therapeutic implications

The distinction between the sensorimotor beta modulation range and the according states of task-related cortico-cortical alpha synchronization may be clinically relevant for two reasons: (i) They may reflect different (patho)physiological mechanisms related to motor and cognitive demands, respectively. (ii) They may be addressed by different neurofeedback interventions. Specifically, neurofeedback of cortico-cortical alpha coherence was recently investigated in healthy subjects and stroke patients with moderate motor impairment; this research showed that such an intervention is feasible and behaviorally useful, thereby providing evidence for the causal role of cortico-cortical synchronization for recovery [Mottaz et al., 2015, Mottaz et al., 2018]. These cortical alpha networks may also be activated indirectly by the very same neurofeedback approach of sensorimotor beta power modulation as applied here. Pilot data of our group in severe stroke with hand paralysis indicated that this approach may also unmask latent corticospinal connectivity when combined with concurrent state-dependent transcranial magnetic stimulation [Gharabaghi et al., 2014a, Gharabaghi, 2015]. Such increased corticospinal connectivity – even when discrete – can be detected, targeted and monitored with refined TMS motor mapping techniques [Kraus and Gharabaghi, 2015, Kraus and Gharabaghi, 2016, Mathew et al., 2016] and will be essential for achieving behavioral gains.

### 6.4 Limitations

While the present study included patients on the basis of the severity of hand paralysis, but with otherwise heterogenous features regarding lesion location, stroke type etc., future studies should consider more homogeneity to avoid potential biases. Only a subgroup of the evaluated patients participated in the subsequent rehabilitation training. Therefore, the observations may be influenced by the low sample size, thus underlining the necessity to confirm the findings in future controlled studies with larger cohorts. Importantly, the present study did not address the specificity of particular therapeutic interventions, which would have necessitated the introduction of a control group. The findings of the present work may rather inform the design of larger future studies; two different approaches may be considered: (i) The presented neurofeedback task may serve as a screening tool to identify good performers and then randomize them to either an intervention or active control group. (ii) The presented neurofeedback task may serve as a prediction tool, while both good and poor performers participate in the same intervention.

Furthermore, the utility of connectivity measures to differentiate those with rehabilitative potential over using simpler measures such as power modulations needs to be addressed with direct comparisons in larger cohorts. In this context, discrete measures, such as the presence or absence of FPI, may be better suitable for clinical application as compared to continuous measures such as power values that necessitate an arbitrary cut-off. Moreover, the effective connectivity measure applied in this study, i.e., the PSI method, is methodologically limited to capture uni-directional information flow only, i.e., information flow in one direction. In cases of bi-directional information flow, i.e., information flow in both directions, either no connection or the stronger uni-directional connection would be detected. Nonetheless, PSI has, already been shown to be a valuable tool for studying effective brain interactions from electrophysiological data [Haufe et al., 2013, Cole et al., 2010], especially for exploratory research questions [David et al., 2004, Stephan, 2004]. When directly comparing PSI with bi-directional coupling methods such as Granger Causality [Granger, 1969], PSI had the advantage of producing less false positive results in physiological data characterized by high noise levels [Nolte et al., 2010]. Unlike measures of connectivity estimated from functional magnetic resonance imaging or diffusion tensor imaging recordings, electrophysiological recordings also provide us with the opportunity for online-assessment of dynamic cortico-cortical coupling, on account of the inherent high temporal resolution of the latter. PSI could therefore be used to inform brain state-dependent stimulation approaches [Raco et al., 2016, Raco et al., 2017, Kraus et al., 2016b, Kraus et al., 2018, Guggenberger et al., 2018].

## 7 Conclusion

This work reveals an association between long-range alpha synchronization and effective sensorimotor beta modulation in the severely lesioned brain. The task-related long-range oscillatory coherence may differentiate severely impaired stroke patients with regard to their rehabilitative potential, a finding that needs to be confirmed in larger patient cohorts.

## CRediT authorship contribution statement

**Kevin Kern:** Conceptualization, Methodology, Software, Formal analysis, Data curation, Visualization, Writing – original draft. **Mathias Vukelić:** Conceptualization, Methodology, Software, Formal analysis, Data curation, Visualization, Writing – original draft. **Robert Guggenberger:** Conceptualization, Methodology, Software, Writing – review & editing. **Alireza Gharabaghi:** Conceptualization, Supervision, Project administration, Funding acquisition, Writing – original draft.

## Declaration of Competing Interest

The authors declare that they have no known competing financial interests or personal relationships that could have appeared to influence the work reported in this paper.

## Data Availability

The authors do not have permission to share data.
